# Adult Upper Cortical Layer Specific Transcription Factor CUX2 Is Expressed in Transient Subplate and Marginal Zone Neurons of the Developing Human Brain

**DOI:** 10.3390/cells10020415

**Published:** 2021-02-17

**Authors:** Terezija Miškić, Ivica Kostović, Mladen-Roko Rašin, Željka Krsnik

**Affiliations:** 1 Croatian Institute for Brain Research, School of Medicine, University of Zagreb, 10000 Zagreb, Croatia; terezija21@gmail.com (T.M.); ikostov@hiim.hr (I.K.); 2 Department of Neuroscience and Cell Biology, Robert Wood Johnson Medical School, Rutgers University, New Brunswick, NJ 08854, USA

**Keywords:** human cortical development, subplate, marginal zone, projection neurons, upper cortical layers, prefrontal cortex, synaptogenesis

## Abstract

Cut-Like Homeobox 2 (Cux2) is a transcription factor involved in dendrite and spine development, and synapse formation of projection neurons placed in mouse upper neocortical layers. Therefore, Cux2 is often used as an upper layer marker in the mouse brain. However, expression of its orthologue CUX2 remains unexplored in the human fetal neocortex. Here, we show that CUX2 protein is expressed in transient compartments of developing neocortical anlage during the main fetal phases of neocortical laminar development in human brain. During the early fetal phase when neurons of the upper cortical layers are still radially migrating to reach their final place in the cortical anlage, CUX2 was expressed in the marginal zone (MZ), deep cortical plate, and pre-subplate. During midgestation, CUX2 was still expressed in the migrating upper cortical neurons as well as in the subplate (SP) and MZ neurons. At the term age, CUX2 was expressed in the gyral white matter along with its expected expression in the upper layer neurons. In sum, CUX2 was expressed in migratory neurons of prospective superficial layers and in the diverse subpopulation of transient postmigratory SP and MZ neurons. Therefore, our findings indicate that CUX2 is a novel marker of distinct transient, but critical histogenetic events during corticogenesis. Given the Cux2 functions reported in animal models, our data further suggest that the expression of CUX2 in postmigratory SP and MZ neurons is associated with their unique dendritic and synaptogenesis characteristics.

## 1. Introduction

The human cerebral cortex is the most puzzling neuronal system of all mammals, which integrate sensory, motor, cognitive, behavioral, and social functions. Underlying these remarkable functions is complex areal, laminar, and columnar organization. This complex organization is established during the prolonged prenatal and perinatal periods characterized by a series of intricate neurogenetic and histogenetic events that achieve the pinnacle of their complexity in developing human brain. Early fetal and midfetal stages of the human brain development include crucial histogenetic events resulting in the basic framework of areal, laminar, and regional organization, and connectivity [1,2]. This basic framework includes formation of transient zones like subplate (SP) and marginal zone (MZ) populated by a number of diverse cells, including neurons that will form first neocortical synapses. The neurons in these transient laminar compartments have a major role in forming first neocortical connections, by providing first projections out of the neocortex and receptive fields for the ingrowing axons with their dendrites, guiding the ingrowing afferents, and forming pioneering and transient neocortical synapses. Therefore, these transient neurons are critical for the formation of first cortical circuits that establish the foundation for later acquired neocortical functions [3,4]. Moreover, midgestational SP serves as a waiting compartment for cortical afferents, and as a migratory zone for postmitotic neurons that are on their way to reach their predisposed position in the cortical plate (CP) [2,5]. The close correlation between the changing patterns of transient cortical laminar organization and cellular neurogenetic events is considered a major feature of the embryonic and fetal telencephalic wall [1,6,7]. Given the magnitude of events that concentrate in transient zones, it is not surprising that disrupted development of neocortical projection neurons and/or SP are both associated with a several neurodevelopmental disorders, including autism and epilepsy [2,8,9]. 

One of the most prominent histogenetic events in cortical development is cellular fate determination, achieved in a process involving molecular and cellular specification of cortical cells [6,10,11,12]. Developing cells have to commit to either neuronal or glial fate after they are born from respective progenitors. After that, newly born neurons will migrate to their final place to finalize the fate specification as well as to differentiate in the laminar-specific cellular phenotypes of the neocortex [13]. It is well known that molecular identities and final fate of distinct neocortical neuronal subtypes are initially determined by the birth date [1,14]. Interestingly, SP seems to be the site of fate selection for cortical deep projection glutamatergic neurons [15,16]. However, neocortical neuronal identities are not completely resolved till the end of midfetal cortical development and their final identity stays plastic during early postmitotic differentiation [17,18]. 

One way to classify neocortical neuronal subpopulations is by selective expression of transcription factors (TF) [10,19,20,21]. TFs take part in molecular specification during the final positioning of cells in the cortical laminae [19,22,23], and are key players in cortical development. In this study, we focused on a TF called Cut-Like Homeobox 2 (CUX2), a member of the cut-like homeobox (CUX) family which has been shown to mediate development and molecular specification of adult upper cortical layers [24,25]. CUX2 is uniquely expressed in the nervous system of murine embryos and adults, acting as a transcriptional repressor suggesting its role in acquiring the cell fate [26,27,28]. In addition, Cux2 was shown to regulate dendrite development, spine development, and synapse formation of projection neurons placed in mouse upper neocortical layers, while CUX2 mutations in humans were associated with generalized epilepsy [28,29]. Collectively, these findings suggest that CUX2 has an important role during neocortical development. However, developmental patterns and laminar shifts of CUX2 expressing neurons, particularly in the transient zones like SP, have not yet been systematically described during human cortical development.

In this study, we analyzed CUX2 expression patterns during laminar development of the human fetal cortex. We propose a potentially novel role for CUX2 in early neocortical circuits and cellular fate selection based on the differential spatio-temporal expression patterns in transient cellular compartments across early and late human fetal cortical development. To date, systematic studies of the spatio-temporal expression patterns of critical modulators of cortical development during human fetal development are scarce. These studies, including ours, are essential for a comprehensive understanding of normal corticogenesis and abnormalities in the neocortex-associated disorders, such as autism and epilepsy [30,31]. 

## 2. Materials and Methods

### 2.1. Human Fetal Brain Tissue

Human brain tissue was obtained during regular autopsies at several clinical hospitals affiliated with the School of Medicine at University of Zagreb. Sampling of tissue was done in accordance with the Declaration of Helsinki (2013), and previously approved by the Internal Review Board of the Ethical Committee at the School of Medicine, University of Zagreb. We used paraffin embedded post-mortem fetal brain tissue from 10 post-conceptual weeks (PCW) to 40 PCW of age (specimens between 28 and 37 PCW were not available for this study), and adult cortical tissue. The fetal age was determined based on the crown-rump length (CRL, in millimeters) and pregnancy records, and was expressed in PCWs. To analyze protein expression during fetal cortical development, at least 2–3 brains per age group were used. Tissue was fixed in 4% paraformaldehyde with adjusted fixation times, dissected coronally in blocks, and processed in alcohol series before being embedded in paraffin. The tissue was sectioned on a microtome (Leica, SM2000R, Wetzlar, Germany) at 8–20 µm thick sections. This study used coronal sections through the cerebral hemisphere at the stratum level, either in its rostral part, or alternatively, the closest midlateral cortex that was available. We took into consideration the discrepancy between somatosensory and visual cortex maturation, where the difference in the visual vs. somatosensory cortex is approximately two weeks [4].

### 2.2. Immunohistochemistry (IHC) and Immunofluorescence (IF) on Postmortem Human Prenatal and Postnatal Cortical Tissue

Prior to proceeding with the IHC/IF protocol, a standard process of deparaffinizing sections was performed in xylol and alcohol series. Antigen retrieval was performed by boiling sections in citrate buffer (pH 6,0). After three washes in PBS, blocking solution containing 1–3% BSA and 0.5% Triton X-100 (Sigma-Aldrich, St. Louis, MI, USA) in PBS was applied on the sections for 1–2 h. The blocking solution was replaced with primary antibodies diluted in a blocking solution and incubated overnight at 4 °C. The following antibodies were used: NeuN (Abcam, Cambridge, UK), MAP2 (Sigma-Aldrich, St. Louis, MI, USA), MAP2 (Merck Millipore, Burlington, MA, USA), CUX2 (Abnova, Taipei, Taiwan), CUX2 (Abcam, Cambridge, UK), Reelin (Millipore), DCX (Merck Millipore, Burlington, MA, USA), FOXP1 (Abcam, Cambridge, UK), Neuroserpin (Abcam, Cambridge, UK), TLE4 (Santa Cruz Biotechnology, Dallas, TX, USA), Nurr1 (R&D systems, Minneapolis, MN, USA). After incubation, sections were washed in PBS, and appropriate secondary antibodies (Alexa Fluor, Thermo Fisher Scientific, Waltham, MA, USA) were applied for 2 h at RT. Following three washes in PBS, TrueBlack quencher (Biotium, Fremont, CA, USA) was applied on the sections. Finally, the sections were covered using a mounting medium with DAPI (Vectorlabs, Burlingame, CA, USA). The IHC protocol was implemented as previously described [32].

### 2.3. RNAscope^®^ In Situ Hybridization

Paraffin-embedded sections were pre-processed for the fluorescent RNAscope^®^ *in situ* hybridization according to the manufacturer’s protocol (Bio-Techne, Minneapolis, MI, USA). The sections were briefly treated with protease for 30 min at 40 ºC, and afterward hybridized for 2 h at 40 ºC with the probe for hCUX2 mRNA (Bio-Techne, Minneapolis, MI, USA). After hybridization, the sections were visualized using the RNA-scope Multiplex Fluorescent Reagent Kit v2 (Bio-Techne, Minneapolis, MI, USA) and the Tyramide Signal Amplification (TSA™, PerkinElmer, Waltham, MA, USA) Plus Cyanine 3. RNAscope^®^ was coupled with immunofluorescence and the next day the sections were incubated with primary antibody MAP2 (Merck Millipore, Burlington, MA, USA) diluted in blocking buffer (2% BSA with 0.5% Triton X-100 in PBS) over night at 4 ºC. Secondary antibodies (Alexa Fluor, Thermo Fisher Scientific, Waltham, MA, USA) diluted in blocking buffer were applied on sections for 2 h at RT. After washing in PBS, the sections were covered using the mounting medium with DAPI (Vectorlabs, Burlingame, CA, USA).

Imaging was performed utilizing a high-resolution digital slide scanner NanoZoomer 2.0RS (Hamamatsu Photonics, Hamamatsu, Japan) and confocal microscope Olympus BX61WI or FV3000 (Olympus, Tokio, Japan), and images were assembled in Fiji and Adobe Photoshop CS6. Laminar distribution was done by counting CUX2 immunoreactive nuclei using the Fiji plugin [33]. Cortical surface area of different coronal sections was segregated into ten bins (Figure 10), which were delineated into the actual compartments where CUX2 was expressed, by comparison to the adjacent Nissl section (MZ, CP, SPu, SPd, IZ, SVZ during early fetal period; MZ, CP, SP during the midgestation; L1–L6, SPrm during the late fetal period).

## 3. Results

We systematically analyzed the neocortical *CUX2* mRNA expression and CUX2 protein expression across all fetal cortical developmental phases, with the special emphasis on SP development: early fetal phase (10–13 CW) during the initial formation of SP, when deep layer neurons are born and first SP circuits are formed; midfetal phase (15–26 PCW) when upper layer neurons are born and migrate through SP, while SP is achieving highest complexity of connections; near-term fetal development when neurogenesis ceased, SP gets resolved, and upper layer neurons reached their positions; and in the adult neocortex. These temporal grouping of results are based on the significant events in SP development as previously defined by Kostovic and Rakic in human and monkey cortical development see also Duque et al. 2016) [4].

### 3.1. Early Fetal Development (10–13 PCW, Pre-SP & SP in Formation Phase)

**Figure 1:**
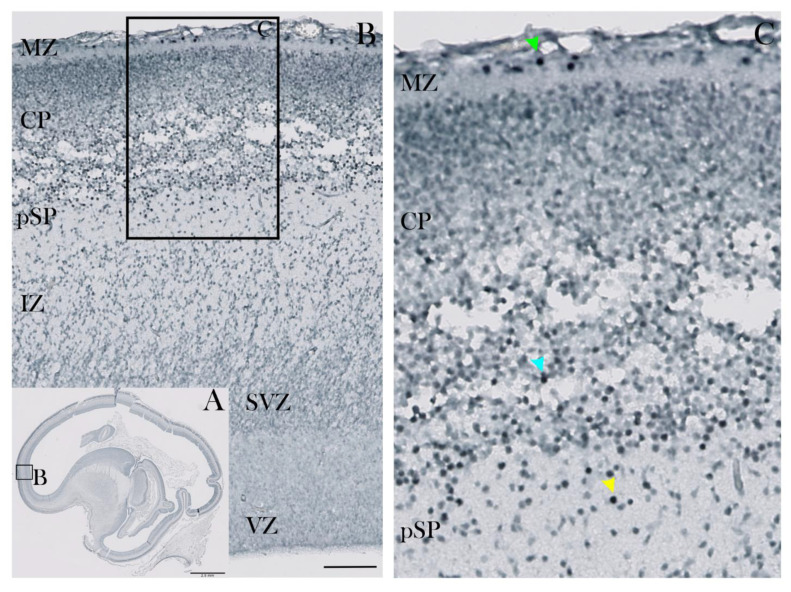
CUX2 is expressed in MZ, deep CP and pSP during the pre-subplate phase at 10 PCW. (**A**) Complete scan of the parasagittal section through the telencephalon of the human fetus immunostained for CUX2. (**B**) Higher magnification light microscopy images of box denoted in A showing developing neocortical wall from the surface-placed Marginal Zone (MZ) to proliferative Ventricular Zone (VZ). (**C**) Parasagittal section through the telencephalon of the human fetus at 10 PCW was immunostained for CUX2. (**C**) CUX2+ nuclei were found in the MZ the deep Cortical Plate (CP; blue arrowhead) and pre-Subplate (pSP; yellow arrowhead), with strongly reactive nuclei in the MZ (green arrowhead). SVZ, Subventricular Zone; IZ, Intermediate Zone. Scale bar = 100 µm (**A**).

**Figure 2:**
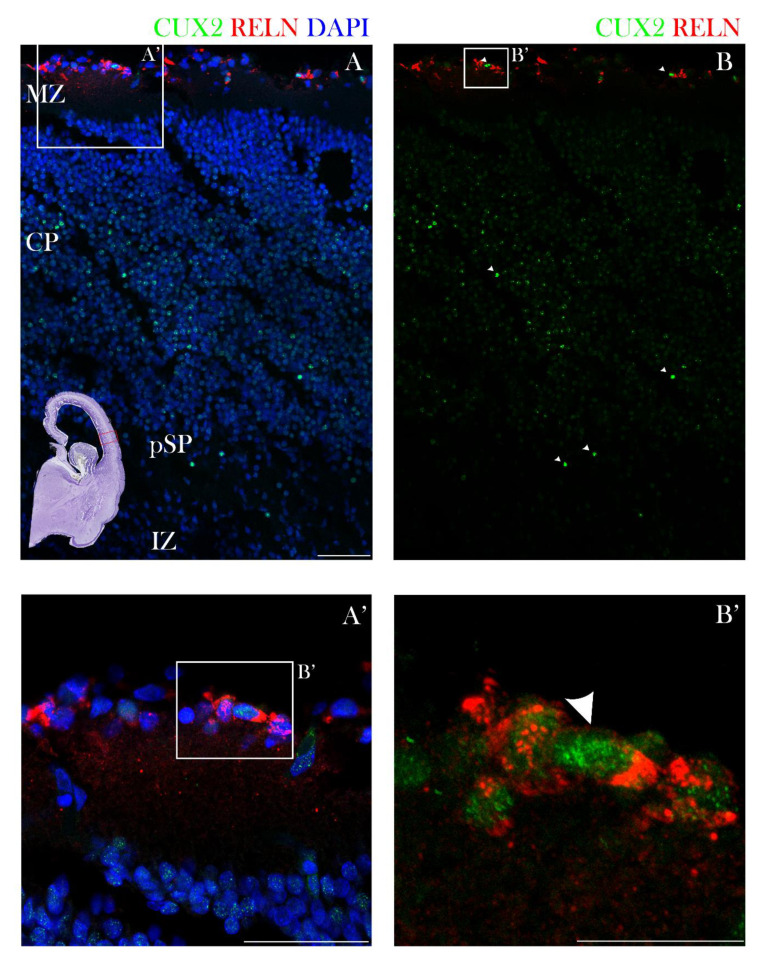
CUX2 co-localizes with Cajal-Retzius neuron subtype marker Reelin in the MZ during the pSP phase at 12 PCW. (**A**,**B**) Midlateral portion of a prospective precentral region of the developing cerebral wall (indicated by box in the Nissl stained complete scan of the coronal section in the lower left corner) during the pSP phase was immunostained for CUX2 (green), Reelin (red), and DAPI (blue). (**A**) A representative confocal image shows evenly spaced CUX2+ nuclei were located in the MZ, while CUX2 was not detected in proliferative VZ and SVZ. (**B**) Reelin co-localized with CUX2+ nuclei in the upper third of the MZ (magnified in **A’** and **B’**). Scale bar = 50 µm.

**Figure 3:**
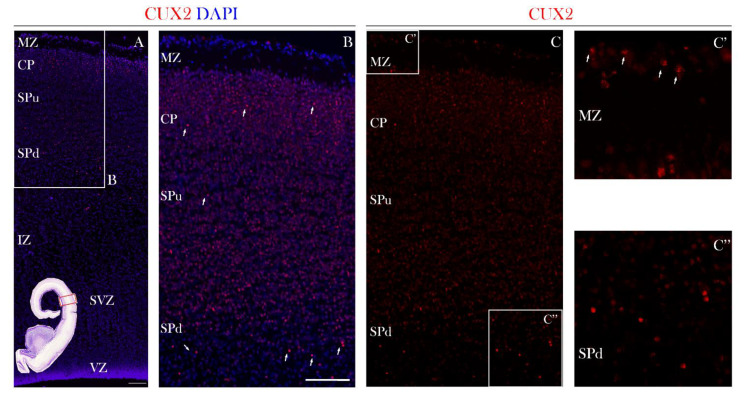
CUX2 is expressed in the MZ, superficial CP, and both upper and deep SP during the SP formation phase at 13 PCW. (**A**,**B**,**C**) Representative images of coronal section through the prospective dorsolateral frontal cortex (indicated by box in the Nissl stained complete scan of the coronal section in the lower left corner), was immunostained for CUX2 (red) and DAPI (blue) during formation of the SP phase at 13 PCW (**C**) CUX2+ nuclei (white arrows) were found to be scattered in the MZ (**C’**), superficial CP, upper SP (SPu; SP in formation), and had strong reactivity in the deep subplate (SPd) (**C’’**).

In the early fetal phase, at 10 PCW, CUX2-protein immunopositive (CUX2+) nuclei were found during formation and first condensation of the cortical plate (CP). In particular, strong CUX2+ nuclei were depicted in the MZ, deep CP and pre-subplate (pSP) (Figure 1). At 12 PCW, CUX2+ nuclei were present in the upper third of the MZ co-localizing with Reelin, a Cajal-Retzius (CR) neuron subtype marker. Heterogeneous and somewhat weaker CUX2+ nuclei were identified within the deepest portion of the CP, while strong reactive nuclei were seen within the pSP of the midlateral cortex that delineates the prospective frontal cortex (Figure 2). At 13 PCW, when the SP is in the process of formation, CUX2+ nuclei were present in both the upper and deep SP, while modest immunoreactivity was observed in the CP and MZ of the prospective frontal cortex (Figure 3). Overall, during the early fetal phase, we did not observe CUX2 reactivity in the germinal proliferative zones, such as Ventricular (VZ) and Subventricular zone (SVZ).

### 3.2. Midfetal Development (15–26 PCW, Stationary SP Phase, SP Expansion Phase Leading to the SP Peak)

**Figure 4:**
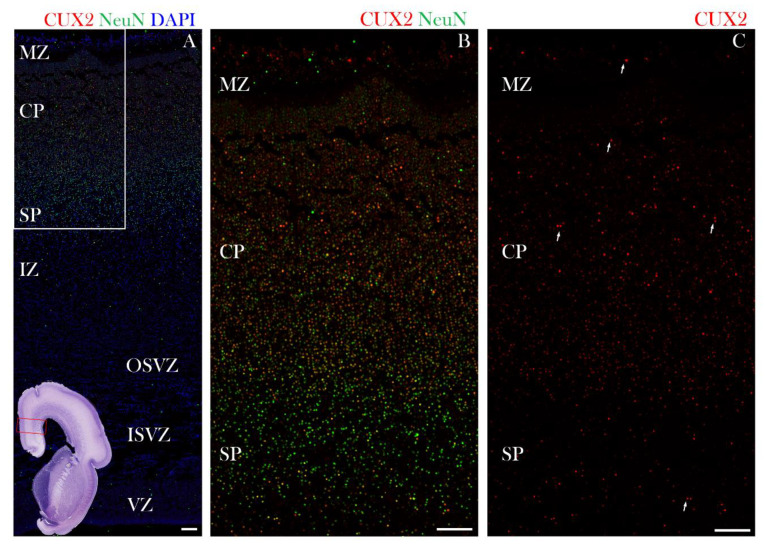
CUX2 is expressed in the MZ, CP and SP during the SP expansion phase at 17 PCW. (**A**,**B**) Coronal section of developing neocortex (indicated by red box in the Nissl stained complete scan of the coronal section in lower left corner), was immunostained for CUX2 (red), neuronal marker NeuN (green) and DAPI (blue) at 17 PCW. In the medial portion of the developing frontal cortex (red box on the Nissl overview image in the lower left corner shows where the images were taken), during the expansion of the SP at 17 PCW. (**B**,**C**) Representative higher magnification confocal images of box A in upper left corner. Strong CUX2+ nuclei were found in the upper half of the CP. In the lower half of the CP, weaker CUX2 nuclear reactivity was present, along with scattered CUX2+ nuclei in the SP and MZ (white arrows mark some of the CUX2+ nuclei). CUX2+ nuclei co-localize with NeuN reactive nuclei. OSVZ, Outer Subventricular Zone; ISVZ, Inner Subventricular Zone. Scale bar = 100 µm.

**Figure 5:**
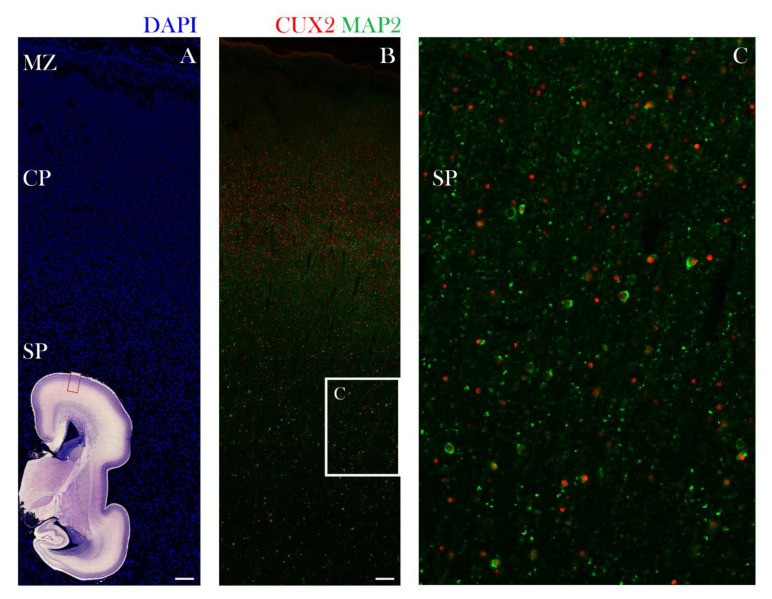
CUX2+ nuclei co-localize with MAP2 immunopositive neurons in the SP during the midgestation stationary phase at 21 PCW. Representative confocal images of the lateral dorsal part of the cerebral cortex in the prospective precentral area (indicated by red box on the inset representing complete scan of coronal Nissl section shown in lower left corner) is shown on the following sections (**A**,**B**,**C**). Developing neocortex was immunostained for CUX2 (red), neuronal marker MAP2 (green) and DAPI (blue). (**A**,**B**) The cell dense CP (A) contains CUX2+ nuclei in the mid third of the CP (**B**). MAP2 co-localizes with CUX2+ nuclei during the midgestation phase in the MZ, CP, and SP. (**C**) Higher magnification of box in B shows that a portion of MAP2+ neurons are also CUX2+ cells in the SP (**C**). Scale bar = 100 µm.

**Figure 6:**
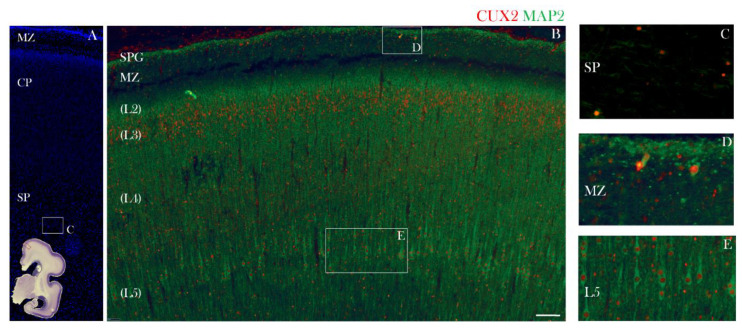
CUX2 is expressed in upper MZ, upper CP, and SP during the peak SP stage at 24 PCW. (**A–E**) Representative images of medial dorsal part of the cerebral cortex in the prospective precentral gyrus indicated by red box on the inset representing complete scan of coronal Nissl section shown in lower left corner of A. (**A–C**) Developing neocortex was immunostained for CUX2 (red), neuronal marker MAP2 (green) and DAPI (blue). During the stationary SP phase at 24 PCW in the prospective precentral gyrus, CUX2 reactivity is most prominent in the upper portion of the CP (**A**,**B**), and SP (**A**,**C**). C is higher magnification of box denoted in A above the inset. D and E are higher magnifications of boxes denoted in B, respectively. Strong CUX2+ reactivity in the MZ (**D**) relates to prospective CR cells, while moderate reactivity in the MZ relates to small subpial granular layer (SPG) nuclei. The giant layer 5 pyramidal cells that are MAP2+ show weak CUX2 reactivity (**B**,**E**). Scale bar = 100 µm.

**Figure 7:**
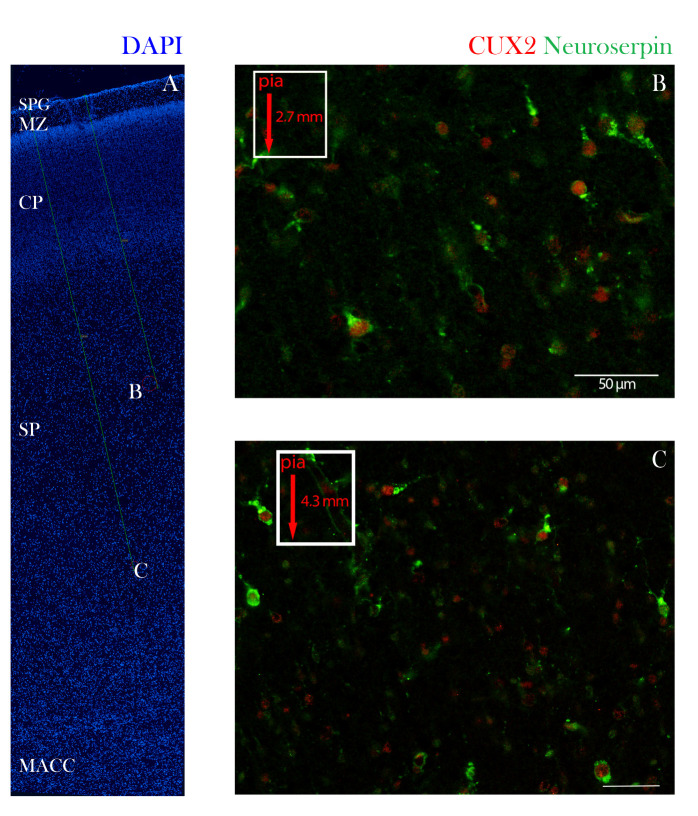
CUX2 is expressed in Neuroserpin expressing SP neurons at peak of SP stage at 24 PCW. Coronal section of developing dorsolateral neocortex was immunostained for CUX2 (red), SP marker Neuroserpin (green) and DAPI (blue) at 24 PCW. (**A**) Red circles denote position of images taken in B and C. A green line demarks the depth from the pial surface. (**B**,**C**) CUX2+ and Neuroserpin co-expression in the superficial SP (B; 2.7 mm from pia, (**B**) and deep SP (**C**); 4.3 mm from pia). Scale = 50 µm (**B**,**C**).

**Figure 8:**
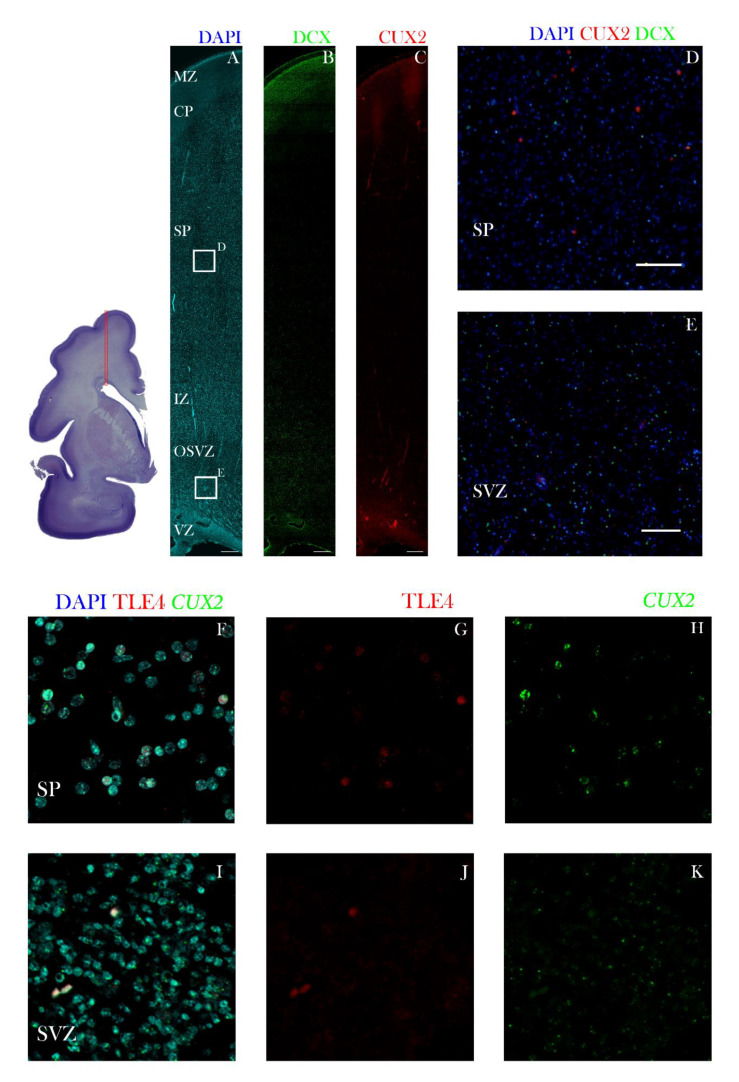
CUX2 is expressed in MZ, CP and SP during the peak of SP at 26 PCW. Top left shows complete scan of frontal coronal section stained for Nissl. Red box denotes place of representative confocal images shown in A–C. (**A–E**) Cerebral wall of the superior frontal gyrus at 26 PCW stained for CUX2 (red), marker of immature migratory neurons DCX (green), and DAPI (blue). D is higher magnification of superficial box denoted in A. E is higher magnification of lower box denoted in A. CUX2+ nuclei were found in MZ, upper part of CP and scattered along the SP (**A–D**). The large CUX2+ nuclei in the SP mostly do not co-localize with DCX neurons (**D**). Somewhat weaker CUX2+ protein expression was detected in the OSVZ (**E**). (**F–K**) Cerebral wall of the superior frontal gyrus at 26 PCW stained marker of deep layer neurons TLE4 (red) and DAPI (blue). Immunostaining was coupled to the fluorescent RNAScope^®^ to detect CUX2 mRNA (green). CUX2 mRNA is expressed in the SP and SVZ, respectively. We did not detect co-localization of CUX2 mRNA and TLE4 in the SP, while CUX2 mRNA expression was detected in the SVZ, paralleling the protein expression. Scale bars: A, B, C = 500 µm; D, E = 100 µm; F–K = 50 µm.

At the onset of midfetal development, at 17 PCW, CUX2+ nuclei were identified in the MZ, CP, and in the SP of the dorsolateral frontal cortex (Figure 4). Furthermore, at 21 PCW, CUX2+ large nuclei were mainly depicted in the middle of the CP. In some cells, CUX2+ nuclei co-localized with multipolar, large MAP2 positive neurons of the SP (Figure 5B,C). MAP2 is used to label differentiated, mature neurons. At the end of midgestation at 24 PCW, CUX2+ nuclei were located in the MZ, CP, and SP of the precentral gyrus (Figure 6). At 24 PCW, MAP2 and Neuroserpin immunoreactive neurons contained CUX2+ nuclei in the SP neurons (Figure 6 and Figure 7), while Nurr1+ nuclei were not confirmed to co-localize with CUX2 (Figure S1). In addition, MAP2 co-localized with CUX2+ nuclei of large, projection pyramidal layer 5 neurons (Figure 6C,E). When the SP reached its peak at 26 PCW, we found *CUX2* mRNA expression in the SP that did not co-localize with TLE4, a marker of deep layer projection pyramidal neurons. Importantly, a scattered signal of *CUX2* mRNA was found in the SVZ, but not as strong as in the CP and SP (Figure 8). 

### 3.3. Late Fetal Development (Near Term, SP Resolution Phase)

**Figure 9:**
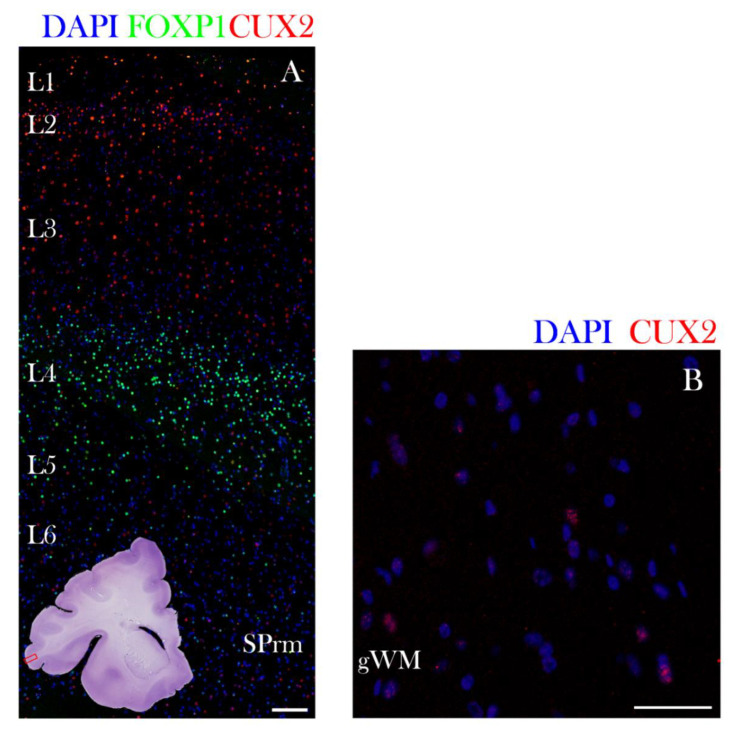
CUX2 is expressed in upper layers at the SP resolution phase at 38 PCW. Representative confocal images of lateral dorsal part of the cerebral cortex in the prospective operculum (indicated by red box on the inset representing complete scan of coronal Nissl section shown in lower left corner of A) are shown on the following sections (**A**,**B**). Developing neocortex was immunostained for CUX2 (red), neuronal subtype marker FOXP1 (green), and DAPI (blue). During the SP resolution phase (38 PCW), inferior frontal gyrus cortex exhibits strong CUX2 expression in layers 1–3, as well as in the SP remnant (SPrm) (A). Gyral white matter (gWM) contains a portion of cells positive for CUX2 (B). Scale bar = 100 µm.

**Figure 10:**
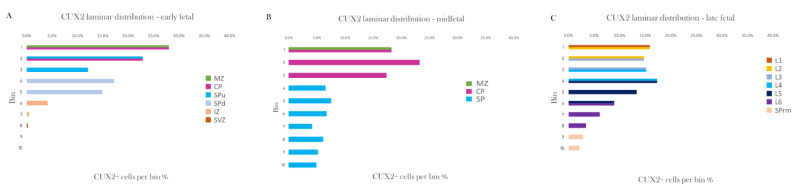
CUX2 laminar distribution presented as a percentage of CUX2 positive cells in the cortical compartments throughout the development. (**A–C**) Cortical surface area of coronal sections was segregated into ten bins, which were delineated into the actual compartments where CUX2 was expressed, by comparison to the adjacent Nissl section (MZ, CP, SPu, SPd, IZ, SVZ during early fetal period; MZ, CP, SP during the midgestation; L1–L6, SPrm during late fetal period).

At the newborn age of 38 PCW, CUX2 had the strongest expression in the upper cortical layer (L1–3) cells and SP remnant (SPrm) in the frontal cortex (Figure 9A; [35]). Furthermore, FOXP1 expressing neuronal subtype of neocortical layers 4 and deep layer 5 had weak CUX2+ reactivity (Figure 9A). Additionally, we found that in the frontal cortex of a near-term, 38 PCW fetus, some of the nuclei of gyral white matter (gWM) maintained CUX2+ reactivity (Figure 9B). In summary, CUX2+ laminar distribution throughout fetal development shows dynamic changes in protein expression while predominantly expressed in differentiated layers of cortex, and later on in the superficial layers (Figure 10).

### 3.4. Adult Period (Post-SP Stage)

**Figure 11:**
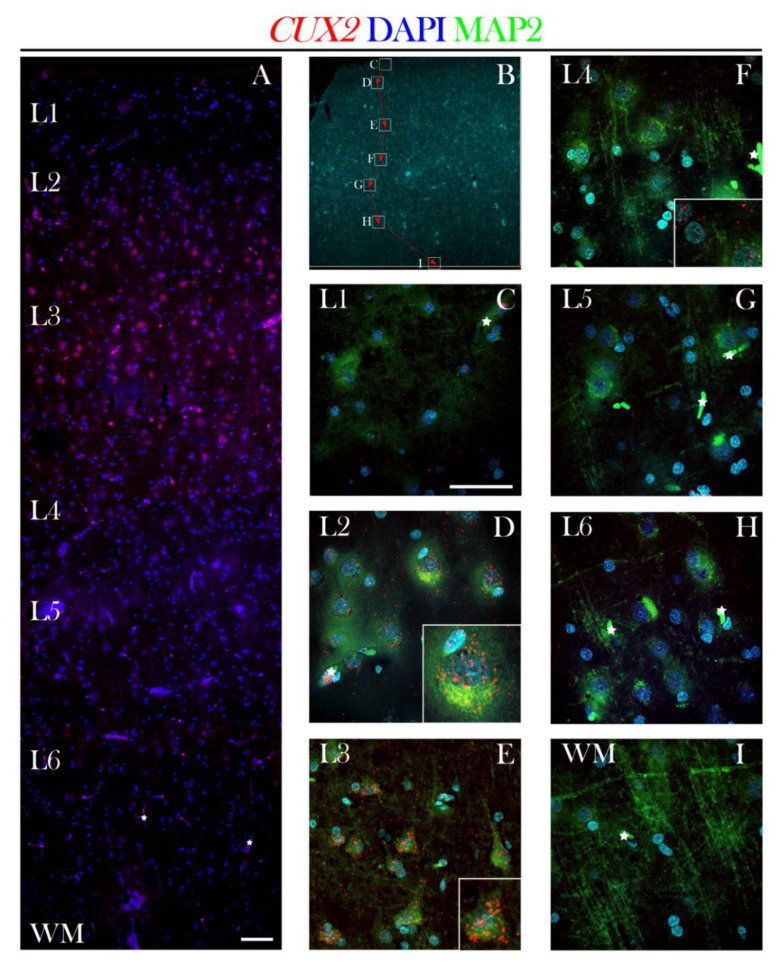
*CUX2* mRNA is expressed in upper layers of adult PFC (18 y). (**A**) *CUX2* mRNA (red) expression in adult PFC in upper layers of the post-SP phase was detected using the fluorescent *in situ* hybridization using RNAScope^®^. (**B**) Overview image of PFC (Brodmann area 9) stained with DAPI. Boxes show respective locations of representative high magnification confocal images taken in C–I. (**C–I**) Single *CUX2* mRNA particles in red are visible in each of the upper cortical layers (L1-4) co-stained with MAP2 (green) and DAPI (blue). White stars depict blood vessels oversaturated with signal. Scale bar A = 200 µm, and C–I = 50 µm.

Using the fluorescent RNAScope^®^ methodology, we confirmed previous reports of *CUX2* mRNA expression in the adult prefrontal cortex (PFC) [36], indicating that CUX2 is exclusively present in layers 2–3. Interestingly enough, *CUX2* mRNA single particles were visualized in soma and neuronal processes of positive neurons, besides the nucleus (Figure 11D,E). *CUX2* mRNA expression was present in layers 2–4, but the strongest signal was seen in layer 3 glutamatergic (pyramidal) neurons, co-labeled with the MAP2 (Figure 11). Furthermore, *CUX2* mRNA was not visible in gyral WM, while possibly non-specific immunoreactive aggregates belong to blood vessels (I). 

## 4. Discussion

The unique identity of developing neurons defined by transcription factors (TFs) dictates neuronal development, their specificity and connectivity, and the ultimate neocortical development. Our results showed that TF CUX2 is expressed in the migratory projection neurons destined for the upper cortical layers during the process of their migration through transient fetal compartments. We also found previously not reported CUX2 expression in the transient populations of postmigratory neurons of the two transient and integral compartments of developing human fetal cortex, the SP and MZ. Our novel findings of CUX2 expression in the transient neuronal populations that do not belong to the upper cortical layers suggest its multifunctional role during prenatal corticogenesis. These two major findings and the possibly diverse roles of CUX2 will be discussed in the following paragraphs of the discussion.

### 4.1. CUX2 in the Developing Migratory Population of the Upper Cortical Layer Projection Neurons 

In this study, we found that during cortical development projection neurons predominantly express CUX2 while migrating through SP and deep CP. These results are consistent with the previous experimental data on rodents [24,25,37] and studies on the post-mortem human brain [38], which showed that CUX2, a marker of upper layer projection neurons is expressed during fetal development. Even though our results are showing scarce reactive nuclei in the superficial intermediate zone (IZ), it is surprising that CUX2 protein or mRNA expression was not present in the subventricular proliferative zone during the neurogenesis, as described in rodents [24,25]. However, in the SVZ of 26 PCW preterm stage, we have visualized limited, weak *CUX2* mRNA and protein expression. Importantly, a period of *CUX2* mRNA expression in the SVZ roughly corresponds to the developmental period in the monkey brain where layer 3 neurons of PFC are born between E70 and E85 [39], and in the visual cortex around E90 [14]. Therefore, we suggest that at least some of the reactivity differences may be attributed to the species difference and the timing of its developmental events. Previously, CUX2, but also CUX1 and BRN2 upper layer markers, were found to be co-expressed with Doublecortin (DCX), a migratory cells marker, suggesting that majority of CUX2+ neurons belong to the migratory neurons in SP [38]. These reports are in line with our findings, collectively suggesting CUX2 role in migration of human neurons.

### 4.2. CUX2 Expression in a Transient Population of Postmigratory Neurons and Early Differentiated Neurons in the Transient Cortical Compartments SP and MZ

The most striking CUX2 expression was featured in the large, Reelin- positive CR cells, and the vast population of polymorphic SP neurons. Even though this reflects the results shown in the mouse cortex [40], we have seen CR cells that are Reelin-positive and have CUX2+ nuclei throughout the fetal development. Our results show CUX2 expression in SP postmigratory neurons in all phases of SP development: SP formation, SP increase, stationary SP, and SP resolution [2,4,32]. The identification of CUX2+ nuclei in SP neurons was based on co-existence with MAP2 and Neuroserpin, markers previously detected in fetal SP neurons [30,41]. 

Moreover, besides migrating upper layer neurons, during the period of midgestation, a part of CUX2+ SP neurons may belong to tangentially migratory GABA-ergic neurons [38]. GABA-ergic neuronal migration has a late-onset [42] and GABA-ergic SP neurons may have long projections [8,43,44]. The rest of the CUX2+ SP population may belong to the glutamatergic population of SP projection neurons. These neurons exhibit transient widespread projections [45]. Indeed, SP neurons project subcortically to the thalamus [46,47], and through the corpus callosum to the contralateral hemisphere [48]. Similar to SP neurons, another population of transient neurons, CR cells, have well-protracted subcortical and intracortical axons and are, thus also considered transient projection neurons [2,49,50]. Thus, we found that beside previously reported CUX2 expression in adult upper layer cortical projection neurons, CUX2 is also expressed in the fetal transient projection neurons. These findings suggest a role for CUX2 in the formation of the neuronal circuits. 

Further and indirect evidence that CUX2 expression is related not only to migratory upper layer neurons but also to the residing postmigratory SP projection neurons is derived from the timing of the CUX2 expression in the SP. First, CUX2 was present in the earliest phase of SP formation, when projection neurons of upper cortical layers are not born yet [14]. Second, CUX2 was expressed in SPrm white matter (WM) neurons around the term age when all neuronal bodies for upper layers are already in their final postmigratory position. Timed decrease of CUX2+ nuclei in CR cells, and SPrm-WM neurons can be explained by the fact that both of these populations, CR and gyral WM neurons, gradually transform their morphology and position themselves in WM [4,51,52,53], possibly losing their projection traits which is their specialized feature for fetal transient networks. Thus, partial loss of CUX2 expression in gWM neurons during postnatal development may be an important indicator of the projection profile of gWM neurons. Due to the extremely rapid growth of WM in the human cerebrum during early postnatal development [7], it is normal to expect changes in the long axonal projection of gWM neurons. However, gWM neurons maintain their local connectivity, serving as ‘gatekeepers’ for afferent inputs reaching superficial gWM [5]. 

Furthermore, a perplexing, but weaker CUX2 reactivity was observed in the pyramidal neurons of prospective layer 5 during midgestation. This finding suggests that the CUX2 role is present during the formation of transient widespread circuits and axonal collateralizations of deepest neocortical layers during development [19,54]. Previously, Cux2 expression was found in deep layers of mouse insular cortex at P5 [37], and in layer 5 of the mouse somatosensory barrel cortex at P14 [55]. Interestingly, a recent human prenatal scRNA-seq study showed evidence of some co-expression between layer 5–6 markers and CUX2 in embryonic and some mid-fetal samples [18], as also seen by qRT-PCR of FACS cells of developing mouse brain [31]. Our findings suggest that the CUX2 neuronal population in the cortex during fetal development is heterogeneous, with a portion of cells migrating toward the CP, and a fraction of cells projecting their axons to specific targets.

### 4.3. Diverse Roles of CUX2

CUX2 expression in multiple neuronal classes at different phases of differentiation suggests its diverse roles in development. Indeed, prior studies in animal models found CUX2 primarily as a layer-specific regulator playing a role in dendritic branching, spine formation and synapse regulation [25,28,56]. Our results show that CUX2 is present in different neuronal populations that are either migrating or are forming first cortical synapses and circuits in transient zones SP and MZ. Collectively, these findings support the idea that CUX2 participates in the molecular specification, dendritic development, circuit formation, and synaptogenesis of fetal transient SP and MZ projection neurons as it does in permanent projection neurons of upper layers. 

In addition, we propose an imminent, much wider role for CUX2 in the telencephalon, related especially to its homeobox gene properties [25]. More specifically, the findings of our study imply that projection neurons have a special role in the large-scale organization of the telencephalon during development, and the presence of CUX2 in transient and permanent projection neurons may be related to this general role. Namely, transient projections across different telencephalic domains provide important signaling for molecular specification of the whole neuronal system, which is necessary in establishing the complex telencephalic organization. This conforms to the prediction that transient projection networks provide both synaptic and non-synaptic signals between remote cerebral regions before final connectivity is established [2]. Finally, CUX2 expression is maintained during adulthood in the upper cortical layers, which suggests its importance for both normal cortical development and the maintenance of predominantly associative and integrative cortical neuronal populations.

Our study provides a molecular and histological characterization of CUX2 neuronal populations within the neocortex during the prolonged period of human cortical development and in the adult. Based on Cux2 roles in other species, we speculate that it can share some of previously identified functions, such as synapse maturation and layer specification. However, being limited to human postmortem tissue, this study lacks axonal tracing or similar experimental approaches to determine the possible projection character of CUX2 neurons. Nevertheless, our finding that CUX2 is expressed in transient but critical SP neurons of neocortical development further stresses the importance of CUX2 mutations associated with neurodevelopmental disorders, such as autism and epilepsy [9]. Future studies in 3D human models should test for both differences and similarities of distinct roles of different transcription factor reported in other species and human brain development.

## Supplementary Materials

The following are available online at https://www.mdpi.com/2073-4409/10/2/415/s1, Figure S1: *CUX2* mRNA is undetectable in Nurr1 expressing SP neurons at 17 and 24 PCW; Figure S2: CUX2 is expressed in DCX migratory neurons at 8 and 38 PCW.
