# Supplementary material for: Adult Upper Cortical Layer Specific Transcription Factor CUX2 Is Expressed in Transient Subplate and Marginal Zone Neurons of the Developing Human Brain
Source: Cells. 2021 Feb 17;10(2):415. doi: 10.3390/cells10020415 (PMC7922267; doi:10.3390/cells10020415)
Supplement: Supplementary file 1 [file cells-10-00415-s001.pdf]

Supplementary figures

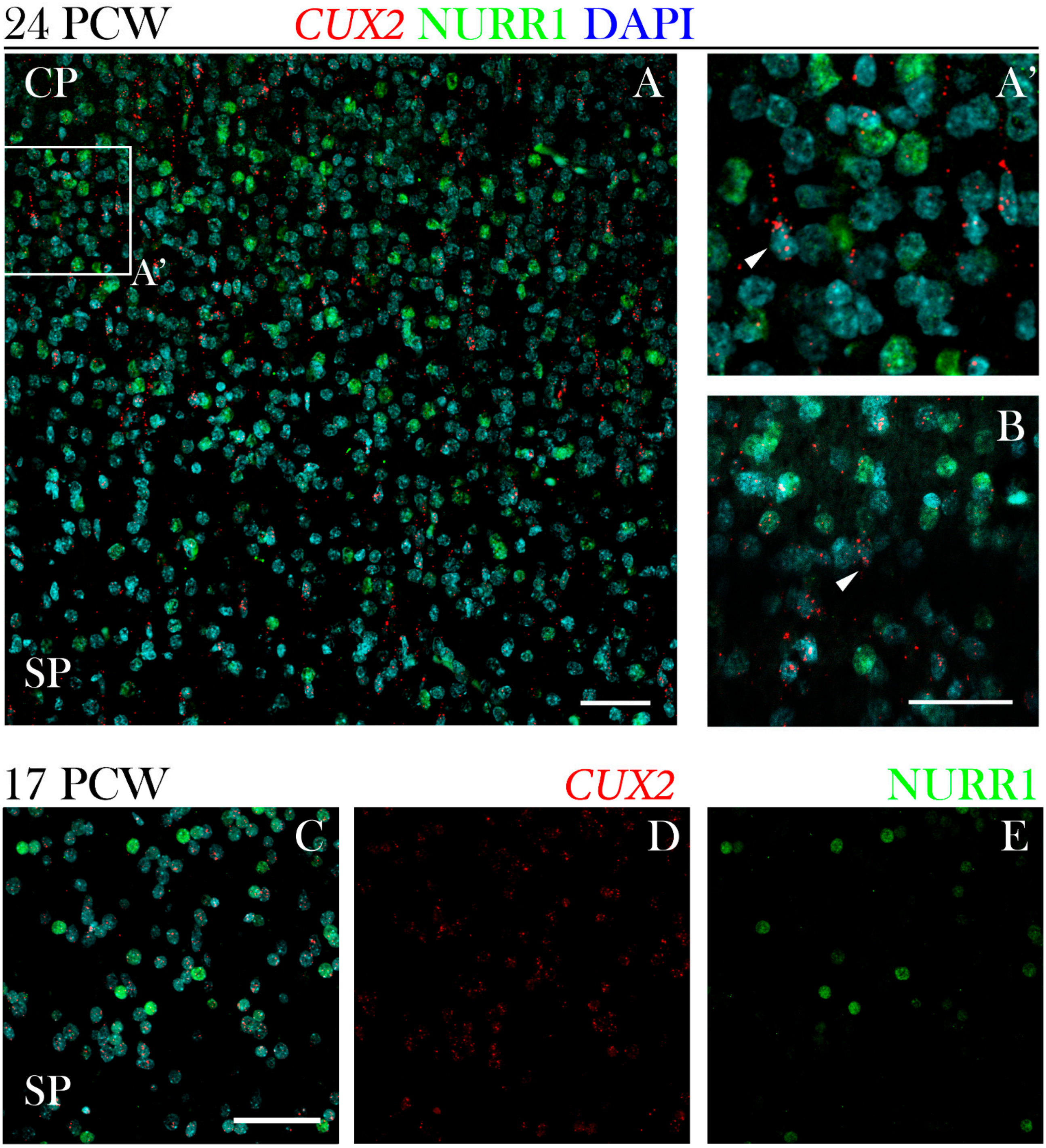

FIGURE S1: ***CUX2* mRNA is undetectable in Nurr1 expressing SP neurons at 17 and 24 PCW.** *CUX2* mRNA (red) was analyzed on coronal section of developing neocortex that were co-

immunostained with SP marker NURR1 (green) and DAPI (blue) at 17 and 24 PCW. Co-expression between NURR1 expressing SP neurons and *CUX2* mRNA was not detected. White arrowheads mark *CUX2* mRNA expressing cell. Scale = 50 µm.

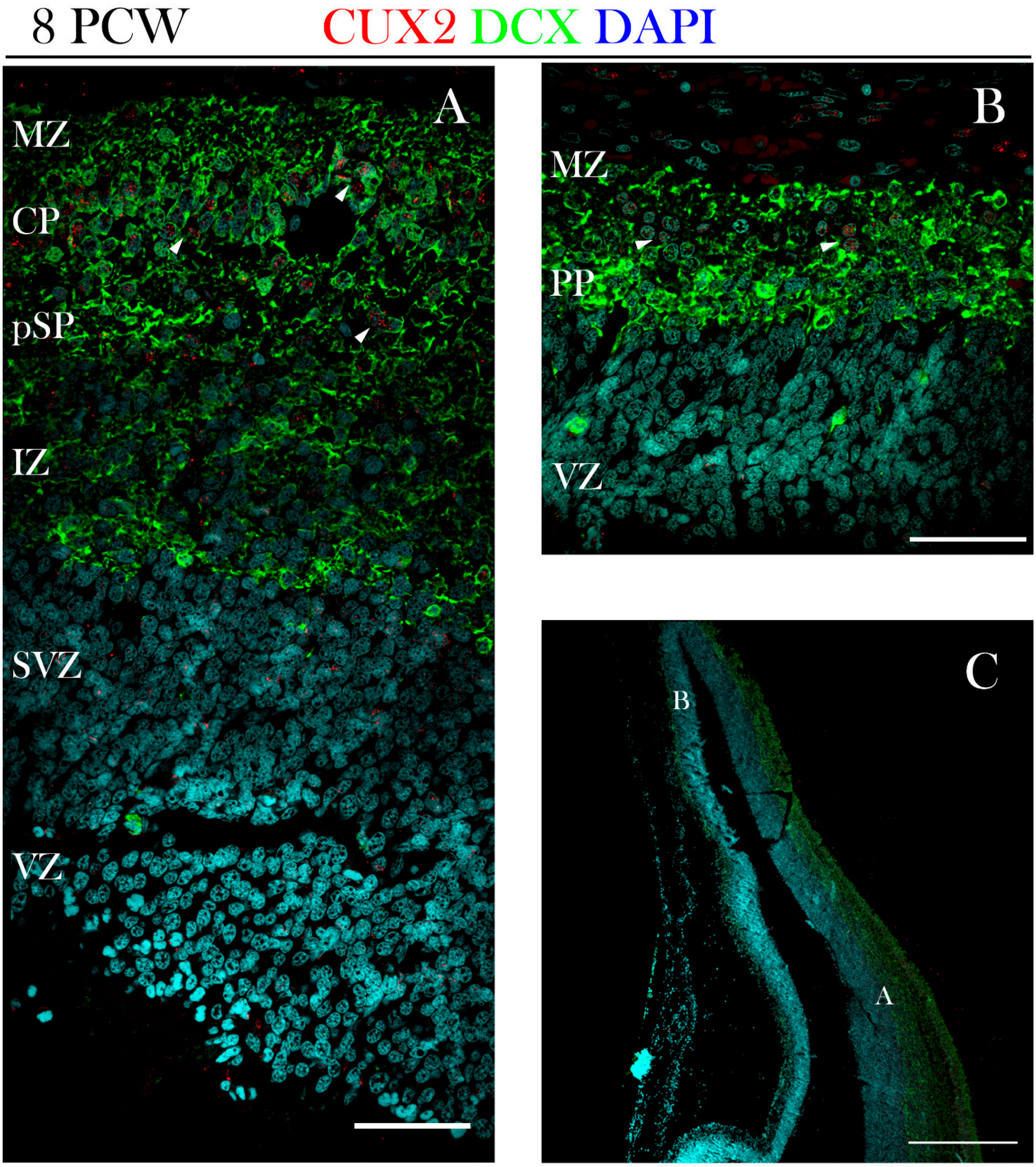

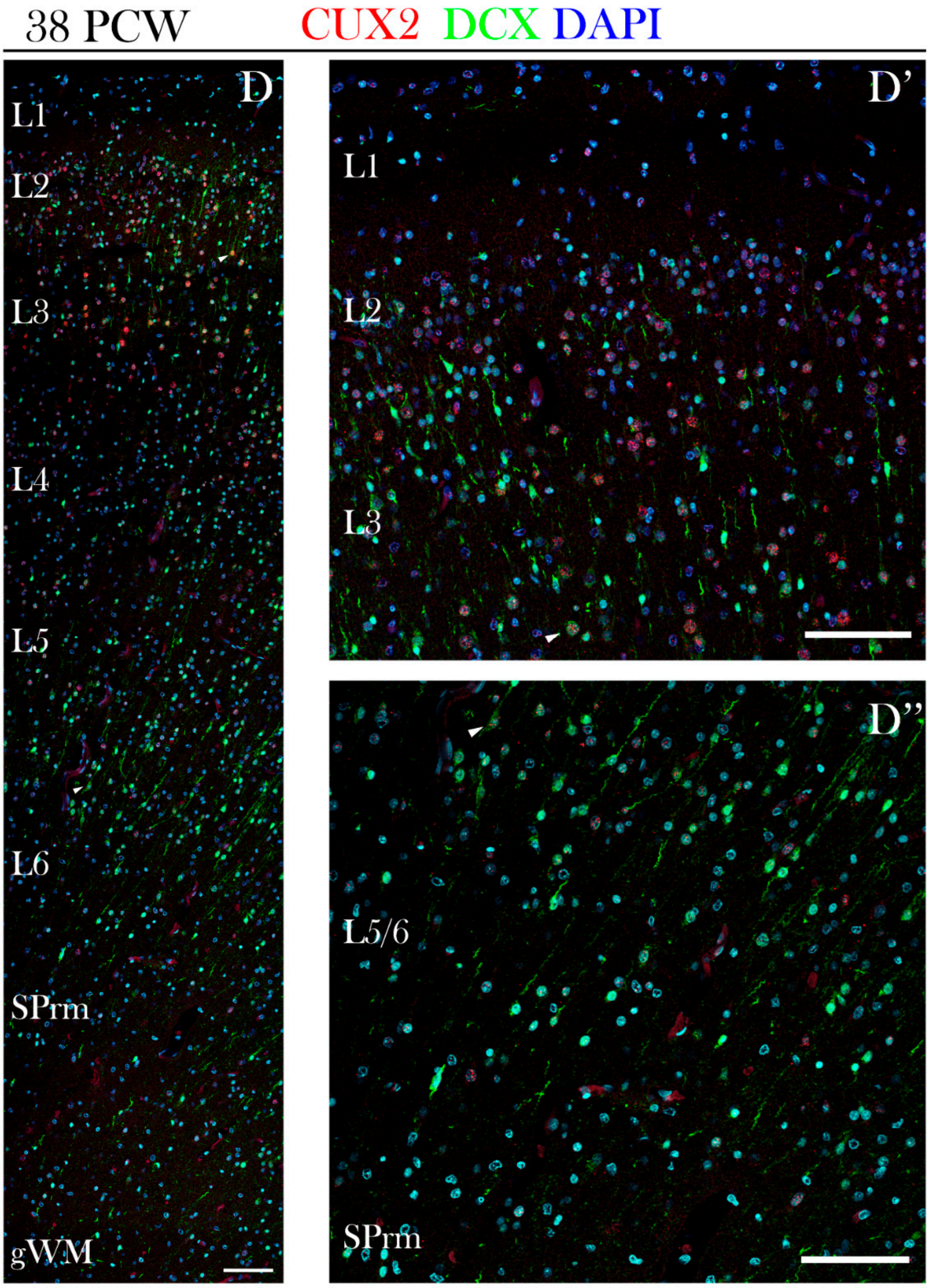

FIGURE S2: **CUX2 is expressed in DCX migratory neurons at 8 and 38 PCW.** Developing frontal neocortex was immunostained for CUX2 (red), marker of migratory neurons DCX (green) and DAPI (blue). CUX2+ nuclei were co-expressed with DCX at 8 PCW (**A-C**) and 38 PCW (**D,D',D''**). CUX2 nuclei (white arrowheads) are depicted in the CP and pSP of ventrolateral cortex (A) at 8 PCW, as well as in the preplate (PP) of dorsolateral cortex (B). Images were taken

in the neocortical anlage as shown in (C). Co-localization of DCX and CUX2 at 38 PCW in the frontal cortex (D). Note that cortical cells especially in the deep cortical layers, maintain DCX expression. Scales = 50 µm (D); 100 µm (C).
